# Formulation of the Polysaccharide FucoPol into Novel Emulsified Creams with Improved Physicochemical Properties

**DOI:** 10.3390/molecules27227759

**Published:** 2022-11-10

**Authors:** Sílvia Baptista, Filomena Freitas

**Affiliations:** 1 Associate Laboratory i4HB-Institute for Health and Bioeconomy, School of Science and Technology, NOVA University Lisbon, 2829-516 Almada, Portugal; 2 UCIBIO-Applied Molecular Biosciences Unit, Department of Chemistry, School of Science and Technology, NOVA University Lisbon, 2819-516 Almada, Portugal; 3 73100, Lda. Edifício Arcis, Rua Ivone Silva, 6, 4° piso, 1050-124 Lisboa, Portugal

**Keywords:** polysaccharide, FucoPol, natural emulsifier, oil-in-water emulsion, experimental design, cosmetics, rheology, texture

## Abstract

Driven by the customers’ growing awareness of environmental issues, the production of topical formulations based on sustainable ingredients is receiving widespread attention from researchers and the industry. Although numerous sustainable ingredients (natural, organic, or green chemistry-derived compounds) have been investigated, there is a lack of comparative studies between conventional ingredients and sustainable alternatives. In this study, olive oil (30 wt.%) and α-tocopherol (2.5 wt.%) containing oil-in-water (O/W) emulsions stabilized with the bacterial fucose-rich polysaccharide FucoPol were formulated envisaging their validation as cosmetic creams. After formula composition design by Response Surface Methodology (RSM), the optimized FucoPol-based emulsion was prepared with 1.5 wt.% FucoPol, 1.5 wt.% cetyl alcohol, and 3.0 wt.% glycerin. The resulting emulsions had an apparent viscosity of 8.72 Pa.s (measured at a shear rate 2.3 s^−1^) and droplet size and zeta potential values of 6.12 µm and −97.9 mV, respectively, which are within the values reported for cosmetic emulsified formulations. The optimized formulation displayed the desired criterium of a thin emulsion system, possessing the physicochemical properties and the stability comparable to those of commercially available products used in cosmeceutical applications.

## 1. Introduction

The global market demand for products based on innovative ingredients and technologies has compelled the cosmetic industry to rapidly increase the research and development of natural, organic, and eco-friendly formulations [1,2,3]. One of the most notorious examples of this growing interest is the incremental utilization of natural polysaccharides in cosmetic formulations. These biopolymers are composed of carbohydrates with several hydroxyl groups that, given their chemical composition, strongly interact with water [1,2]. There are many functional polysaccharides, able to act as film formers, gelling agents, thickeners, suspending agents, conditioners, and emulsifiers. These features derive from the biopolymers’ physical and chemical properties and are critical for polysaccharide-based cosmetics formulation technologies [1]. Examples of natural polysaccharides with consolidated utilization in commercial skin-care products include xanthan gum and cellulose, which are used as thickeners and stabilizing agents, and hyaluronic acid, which is applied as a moisturizing and bioactive ingredient [1,2,4,5,6]. Besides polysaccharides, many proteins have also been demonstrated as good emulsifiers for example in food products [7,8,9]. The emulsifying ability of proteins derives from their amphiphilic character conferred by the presence of hydrophobic and hydrophilic amino acids in their structures, which allow proteins’ adsorption at oil/water interfaces, thus stabilizing the emulsions [10]. However, proteins have low surface activity than most conventional emulsifiers and the final products’ properties are often impaired by the pH, temperature, and ionic strength [11]. To overcome such disadvantages of proteins, they can be combined with other compounds, such as polysaccharides. The combination of polysaccharides with proteins contributes to the stability of the emulsions, in which polysaccharides provide colloidal stability (thickening and gel-like behavior) and proteins form a viscoelastic layer (through oil-water interface adsorption) [7,9].

Emulsions are extensively used in cosmetic products to stabilize active substances, bioavailability, and sensory properties [12]. Being complex multiphase systems, emulsions’ stabilization with polysaccharides is obtained by increasing the viscosity of the aqueous phase, thereby inhibiting droplet movement [12,13]. The high molecular weight and presence of hydrophilic groups often provide polysaccharides with thickening ability and water-holding properties, two attributes of interest for their application in cosmetic formulations [13]. Classical oil-in-water (O/W) emulsions comprise a continuous phase, in which oil is present as a dispersed phase, and emulsifiers stabilize oil droplets dispersion [1,12]. Emulsions’ rheological properties are essential physical attributes of these systems [14]. From the customer’s point of view, the cosmetics’ functional properties are critical: the product cannot be overly fluid (no structure, low viscosity) nor extremely dense (highly structured, high viscosity) [15]. Moreover, the product’s viscosity can influence the mixing, pumping, and packing process [15,16]. Finding the optimum formulation and process conditions is essential during the development of new emulsions systems considering the final product stability, which influences its shelf life [3,15].

FucoPol is a high molecular weight (1.7 × 10^6^–5.8 × 10^6^ Da) bacterial fucose-rich polysaccharide, with a fucose content of 32–36 mol% and nearly equimolar contents of glucose (28–34 mol%) and galactose (25–26 mol%). Its structure also comprises glucuronic acid (9–10 mol%), and acyl groups: acetate (3.5–6.8 wt.%), pyruvate (3.7–14 wt.%), and succinate (0.6–3.0 wt.%) [17]. The development of emulsions based on FucoPol has been widely studied [14,17,18,19,20]. For instance, Baptista et al. [14] developed an innovative O/W emulsion, composed of olive oil and α-tocopherol as the oil phase; FucoPol was used in the aqueous phase and presented a stabilizing effect, which translated into appropriate rheological and textural behavior of the emulsion. In addition, FucoPol has bioactive properties that further sustain its potential for use in the cosmetic field, such as wound healing ability [21], photoprotection [22], and antioxidant effect [23].

In this study, the main objective was the development of O/W emulsions using FucoPol as a substitute for synthetic emulsifying agents commonly used in cosmetic products. RSM was used to define the optimal concentration ranges for FucoPol, cetyl alcohol, and glycerol. FucoPol-based cosmetic formulations were prepared and characterized in terms of physical stability, rheological, and textural properties, and compared with cosmetic emulsion-based products available on the market.

## 2. Results and Discussion

### 2.1. O/W Emulsions’ Optimization

With the objective of defining the composition resulting in emulsions with high EI after 24 h (E24), concomitant with high apparent viscosity, different FucoPol, cetyl alcohol, and glycerin concentrations were tested. High E24 values (≥95%) were obtained in most runs, except in runs 7, 8, 9, and 11, which were devoid of the FucoPol, irrespective of the cetyl alcohol and glycerin content that varied from 0.0–1.5 wt.%, and 1.0–3.0 wt.%, respectively. Higher FucoPol concentrations also conferred higher apparent viscosity to the emulsions, regardless of cetyl alcohol and glycerin concentrations. The emulsions presented a yellowish-white color, olive odor, and creamy/smooth texture, showing physical stability (as shown by the centrifugation test) for apparent viscosity values ≥90 Pa.s. Table 1 shows that the maximum apparent viscosity obtained values were 249 Pa.s (Run 4) and 244 Pa.s (Run 6), both containing a FucoPol concentration of 1.5 wt.%.

ANOVA was used to define the working ranges for each variable resulting in the highest E24 and *ƞ* values. The coefficients of multiple determination (*R*^2^) values of E24 and *ƞ* were 0.974 and 0.995, respectively. For *ƞ*, the *R*^2^ was in reasonable agreement with the adjusted *R*^2^ (0.989) and the predicted *R*^2^ (0.967). The adjusted coefficient of determination indicated that 98.9% of the variability in the response could be explained by the model. The quadratic model was significant (*f*-value = 169.92 and *p*-value < 0.0001), being supported by an insignificant lack-of-fit (*p* = 0.778) toward the response (*ƞ*), meaning that the error predicted by the model was above the error of the replicas [24]. There is only a 0.01% chance for a noise-derived “Model F-Value”, which implies an adequate variation of the data around its mathematical mean; in addition, the estimated factor effects are real [14,25,26]. The statistical analysis indicates that the proposed model was adequate to predict the ingredients’ concentrations to obtain emulsions with higher viscosities. The same did not happen for E24, where the *R*^2^, adjusted *R*^2^, and predicted *R*^2^ were 0.974, 0.941, and 0.718, correspondingly. The difference between the predicted *R*^2^ and the adjusted *R*^2^ was higher than 0.2, which may indicate a large block effect or a possible problem with the model and/or data.

The RSM results (Figure S1) suggest that cetyl alcohol and glycerin did not influence the E24 and *ƞ* values. Moreover, FucoPol at 1.5 wt.% led to emulsions with *ƞ* values above 206 Pa.s. Increasing the concentration of FucoPol resulted in more viscous emulsions, and more stability against coalescence, avoiding emulsions’ phase separation [14,27]. This is due to FucoPol’s ability to avoid droplets creaming and promote an increased viscosity of the formulation, as reported before [14]. Based on these results, the ingredient concentrations that promoted higher *ƞ* and E24 values were defined as: 1.5 wt.% FucoPol, 1.5 wt.% cetyl alcohol and 3.0 wt.% glycerin.

### 2.2. Characterization of the Emulsified Formulations

#### 2.2.1. Physicochemical Characterization

**Figure 1:**
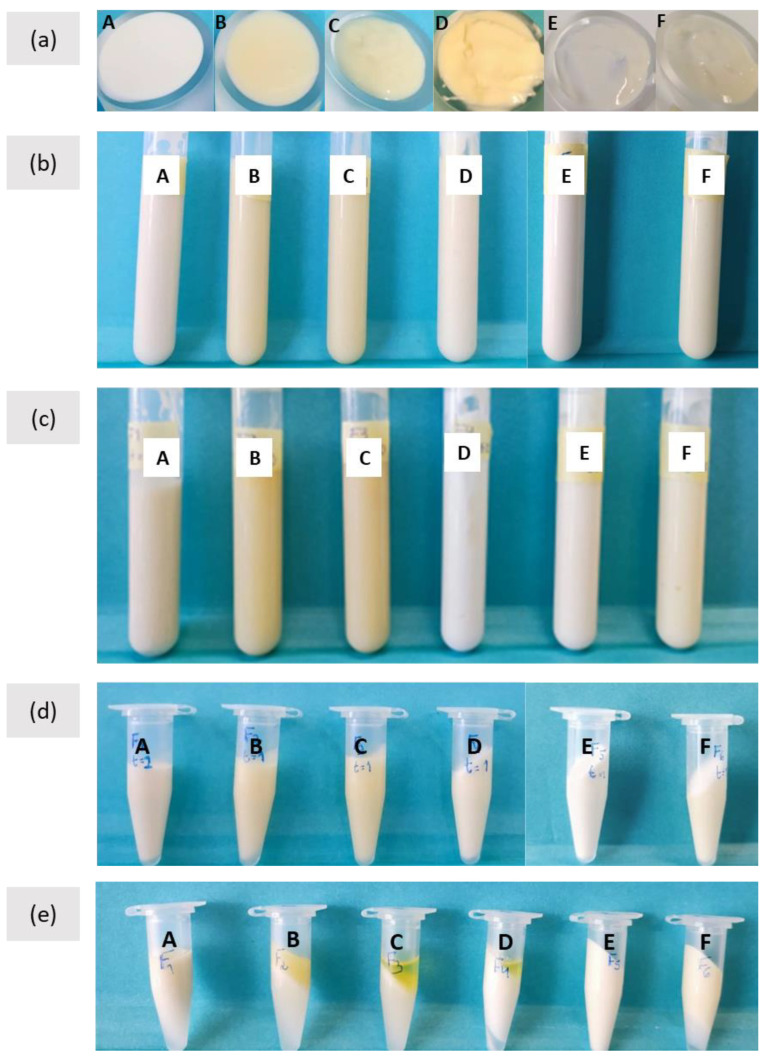
Formulations A, B, C, D, E, F: (**a**) freshly prepared formulations (t = 0); (**b**) after 1 day; (**c**) after 60 days; centrifugation test for 1 day of storage (**d**) and for 60 days of storage (**e**).

The freshly prepared formulations (Figure 1a) presented a yellowish-white color (except formulation A which was completely white) and had a slight olive oil odor. Macroscopic observation, throughout the 60-day storage period (Figure S2), showed the formulations maintained their homogeneous texture, with no visible oil/water phase separation, as confirmed by their EI that was kept unchanged (100%) (Figure 1b,c). The formulations’ physical stability (Figure 1d,e and Figure S3) was evaluated by the centrifugation test to check for the presence of phase separation [26], sedimentation, and/or precipitation [28]. Formulations A, E, and F remained stable for 60 days, showing no phase separation, while formulations B, C, and D showed phase separation at 30 days of storage.

**Figure 2:**
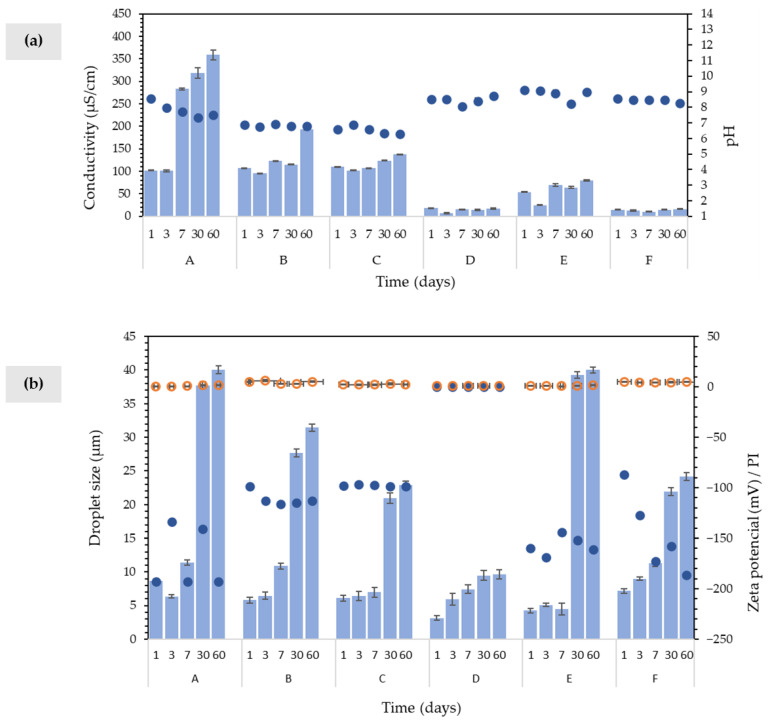
(**a**) pH (circles) and conductivity (bars) and (**b**) droplet size (bars), Zeta-potential (blue circles), and PI (orange circles) for formulations A–F during the storage period (t = 1, 3, 7, 30, 60 days).

As presented in Figure 2a, formulations B and C were slightly acidic with pH values in the range of 6.3–6.9 throughout the storage period (60 days), whilst formulations A, D, E, and F had pH values above 7. Skin care products must not affect the acid–base balance of the skin’s individual layers nor disrupt the *stratum corneum* barrier function [29]. Given the skin’s surface pH (5.5), an acceptable formulation should have a pH value ranging from 4.0 to 7.0 [26,30,31], to avoid skin irritation [32]. Interestingly, the pH value of formulation C (1.5 wt.% FucoPol, 1.5 wt.% cetyl alcohol, and 3.0 wt.% glycerin) was within the optimal range from 6.59 ± 0.01 to 6.30 ± 0.01 during the whole 60-day study period, supporting its suitability for use as a topical cream.

**Figure 3:**
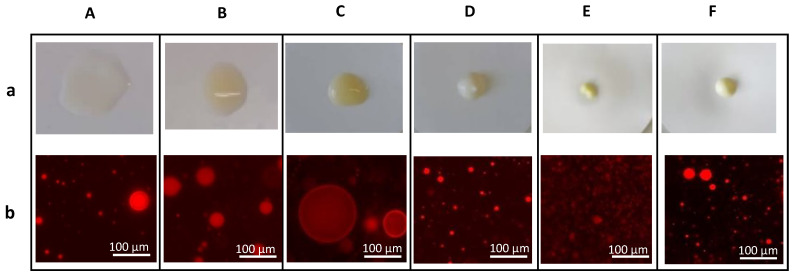
FucoPol-based formulations (A, B, C) and formulations based on stearic acid (D), Sepigel^®^ (E), and stearic/Sepigel^®^ (F): (**a**) Emulsion determination test by filter paper wetting; (**b**) visualization of oil droplets stained with Nile Blue A, under the optical microscope.

The conductivity value, which is indicative of the number of free ions and water present in the system [26], is used to detect physical modifications [33] and to assess if the formed emulsion is an O/W or a W/O system [31,34]. As observed in Figure 2a, formulation A showed a significant increase in the conductivity value (from 102 ± 0.6 to 283 ± 2.0 µS/cm) after 7 days of storage, while for formulations B and C the changes were less significant (from 106 ± 0.3 to 122 ± 0.2 µS/cm, and from 109 ± 0.9 to 107 ± 0.7 µS/cm). Conductivity stability over the 60-day storage period (Figure 2a) indicated an absence of physical changes for formulations C, D, and F. Formulations A, B, and C presented higher conductivity values (>100 µS/cm) corresponding to an O/W system, indicating that the aqueous phase is the continuous phase of the system, whereas the oil phase is nonconductive [34]. Formulations D, E, and F (<50 µS/cm) are considered W/O systems. This result corroborates the emulsion determination test (Figure 3a), and the microscopic observation (Figure 3b), where formulations A, B, and C droplets dispersed on the filter paper, thus confirming their O/W nature [14,35,36]; and showed compartmentalized structures characteristic of O/W systems, consisting of dispersed oil droplets in the aqueous phase [14,37]. Thus, these results confirm that FucoPol forms O/W emulsions, in contrast to Sepigel^®^ 305 and stearic acid under the same conditions. In addition to acting as an emulsifying agent, FucoPol appeared to have a pH-lowering effect. Consumers prefer O/W emulsions due to their sensorial properties (easy to spread, non-greasy) [14,38,39] representing nearly 65% of the total emulsified products available in the cosmetic industry [16].

The formulations’ physical stability was also assessed by measuring the droplet size during the storage period at room temperature (~20 °C). The distribution profile of oil droplets and their size influences the emulsion’s stability, with smaller droplet sizes and lower PI values (<0.3) being responsible for higher stability [3,26,40,41,42]. As shown in Figure 2b, all formulations presented a droplet size characteristic of macroemulsions (>0.1–50 µm), experiencing a considerable increase in droplet size after 30 days of storage. This effect was less evident for formulation D (3.17–9.63 µm), which contained a higher concentration of stearic acid (5.0 wt.%) compared to formulation F (1.5 wt.% stearic acid), which suggests that higher emulsifier concentration allows a decrease of the droplet size and, consequently, increased stability during storage [43]. At lower emulsifier concentrations, the droplet covering ability of the emulsion decreases, causing the coalescence of neighbor droplets that results in the formation of larger droplets [44]. Furthermore, non-ionic emulsifiers can reduce the droplet size of olive oil (triglycerides)-in-water emulsions [45]. For FucoPol-containing formulations, the addition of cetyl alcohol and glycerin (formulation C, Figure 2b), allowed for a decrease in the droplet size (8.68–40.0 µm to 6.12–24.2 µm) and a slight increase of the stability during storage, when compared to formulation A. In general, the droplet size of an emulsion is determined by the homogenization technique applied, the environmental conditions, and the ingredients used for its preparation [46]. Furthermore, there are some technical issues to obtain small droplet-size emulsions using polysaccharide-type emulsifiers [6]. The ideal monodisperse system should have a PI value lower than 0.3 [34,41], which was not verified in any of the formulations (0.47 ≤ PI ≤ 5.02 for t = 60 days) indicating considerable polydisperse droplet sizes.

As shown in Figure 2b, the Zeta-potential of formulations A, B, C, E, and F was −193 mV, −98.4 mV, −97.9 mV, −160 mV, and −86.7 mV, respectively: with evident stability for formulation C during the storage period. The formulation is considered stable when the Zeta-potential value is more than +25 mV or lower than −25 mV [26]. However, some W/O emulsions are highly stable despite having low Zeta-potential values [47], such as formulation D, which showed a rapid aggregation regardless of its absolute Zeta-potential value (0.0 mV) [48].

#### 2.2.2. Rheological Assessment

**Figure 4:**
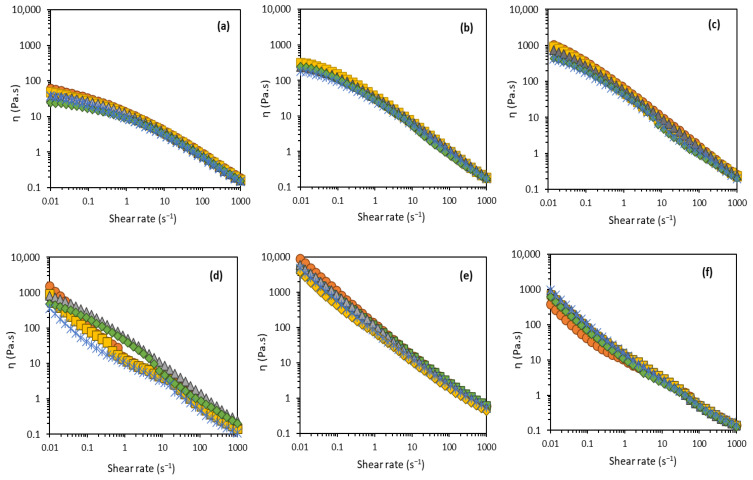
Flow curves for the prepared formulations: A (**a**), B (**b**), C (**c**), D (**d**), E (**e**), and F (**f**) during the storage time; t = 1 day (orange), t = 3 days (yellow), t = 7 days (gray), t = 30 days (green), and t = 60 days (blue).

All formulations exhibited a similar shear thinning behavior to the torque response, as the viscosity gradually decreased under increasing shear rates (Figure 4). The viscosity decrease under a shear rate is attributed, in shear thinning emulsions, to their semi-flexible molecular structure [49]. Except for Formulation E (Figure 4e), all formulations exhibited a slight decrease in viscosity during the storage time. As mentioned before, formulations containing stearic acid became hard during storage, corroborated by the increase in viscosity values over the storage time.

Cosmetic preparation stability over storage time is related to its tendency to exhibit changes in particle migration [29]. In fact, for the FucoPol-based formulations (Figure 4a–c, Table 2), compared to formulation A (8.7 Pa.s), there was an increase of the viscosity to 19.5 Pa.s in formulation B with the addition of 1.5 wt.% cetyl alcohol (Figure 4b); in formulation C (Figure 4c) the addition of both glycerin (3.0 wt.%) and cetyl alcohol (1.5 wt.%) further increased the viscosity to 34.3 Pa.s. This demonstrates that, contrary to the result obtained in Section 3.2, glycerin and cetyl alcohol led to increased apparent viscosity. This may be due to the homogenization method applied (mechanical homogenization vs. manual homogenization) or to the upscale, from 5 g to 100 g, which possibly changes the behavior and efficiency of the ingredients [3]. Comparing formulations C (34.3 Pa.s) and F (6.2 Pa.s) (Table 2), it is possible to conclude that, for the same emulsifier concentration (1.5 wt.%), FucoPol conferred significantly higher apparent viscosity than stearic acid.

**Figure 5:**
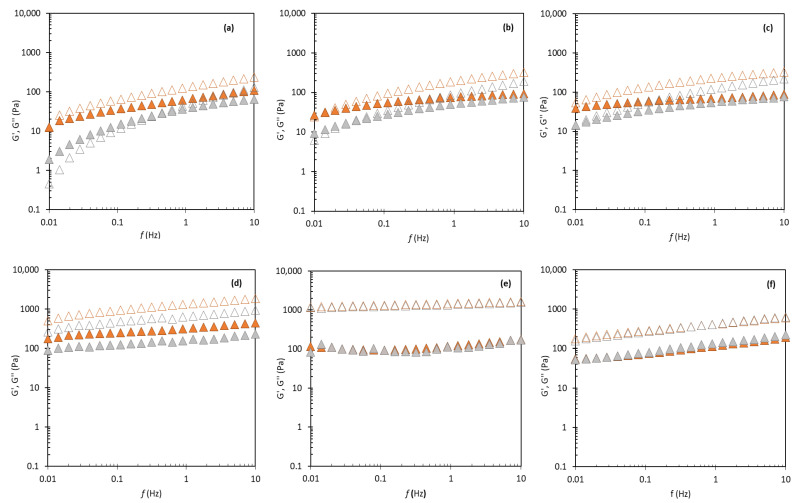
Mechanical spectrum for Formulations A (**a**), B (**b**), C (**c**), D (**d**), E (**e**), and F (**f**), at 1 day (orange) and 60 days (gray) of storage: G′ (open triangle) and G″ (closed triangle).

As shown in Table 2, all formulations showed solid-like behavior, with the storage module higher than the loss module (G′ > G″ at 0.1 Hz). This behavior was more pronounced in formulations D, E, and F, meaning that these formulations present a strong network [15,49,50] with higher stability. Formulations A, B, and C showed a weak gel rheological pattern with an increasing difference between G′ and G′′ values as the frequency increases from 0.01 to 10 Hz. This behavior indicated a dominance of the elastic components over the viscous components of the system, and that physical bonds between the macromolecules held the system’s structure [51]. Figure 5 illustrates the structural stability for the first day and after 60 days of storage at room temperature. The formulations containing a synthetic emulsifier showed much higher values of G′ and G″ than FucoPol. The G′ and G″ modules of most formulations decreased with storage time, except for formulations E and F, suggesting more structured systems, which can influence the spreading behavior [3]. For formulations A and B, it was visible a crossover at 0.01 Hz at t = 1 day, while for t = 60 days the crossover occurs at higher frequencies (0.3 Hz for formulation A, and 0.03 Hz for formulation B). For formulation C, G′ gradually became bigger than G″ during the whole frequency range investigated (0.01–10 Hz) (see supplementary material, Figure S4). Similar behavior has been reported for bacterial cellulose emulsions [50].

#### 2.2.3. Textural Assessment

The textural parameter values (firmness, consistency, cohesiveness, and adhesiveness) of the prepared emulsified formulations are summarized in Table 3. In general, at the end of the storage time (60 days), a decrease in the firmness, consistency, and adhesiveness of the formulations was observed. However, there are some relevant considerations for the FucoPol-based formulations: the addition of glycerin and cetyl alcohol increased not only their apparent viscosity but also their firmness and cohesiveness. In fact, the addition of cetyl alcohol increased the firmness from 0.064 N (formulation A) to 0.162 N (formulation B), while further adding glycerin (formulation C) resulted in increased firmness (0.194 N). These results are concordant with the *η* values (Table 2), where formulation A exhibited lower apparent viscosity (8.72 Pa.s) than formulation C (34.3 Pa.s).

Spreadability is an important texture parameter that infers on the product’s contact with skin (i.e., how it feels on the touch) and ease of removal from packaging, which may affect utilization compliance [52,53]. This parameter is crucial in cosmetic emulsion development being a decisive factor for consumers’ approval of products [14,54]. Formulation A at t = 1 day showed lower firmness (0.064 N) and consistency (0.261 mJ) values, indicating a more spreadable cream sample [14]. On the other hand, formulations C and E showed lower spreadability than the others. Consistency, a textural parameter directly influenced by viscosity, determines the cosmetic formulation application on the skin (higher consistency means a higher difficulty of application and vice-versa) [29]. In terms of adhesiveness, formulations B (0.467 mJ), C (0.387 mJ), and E (0.499 mJ) seemed to be more adhesive than formulations A (0.244 mJ), D (0.338 mJ), and F (0.317 mJ). For FucoPol-based formulations, glycerin and cetyl alcohol positively impacted the physical characteristics. These results are consistent with the rheology assays.

### 2.3. Comparison of FucoPol-Based Formulation with Commercial Cosmetic Creams

Formulation C (after 60 days of storage) was compared to several cosmetic products available in the market in terms of pH, conductivity, droplet size, physical stability (by centrifugation test), and rheological and textural parameters. In the centrifugation test to assess the physical stability, both Formulation C and Sephora^®^ hand cream showed phase separation. As shown in Table 4, Formulation C presented a pH value similar to Uriage^®^ Xémose (face cream) (6.68) but lower than the other tested commercial products, such as Shiseido^®^ primer (8.17) and Sephora^®^ hand cream (8.18). These values are higher than the optimal pH range (between 4.0 and 7.0) compatible with human skin. Nonetheless, the droplet sizes of Shiseido^®^ primer (22.0 µm) and Sephora^®^ hand cream (27.9 µm) are very similar to that of Formulation C (24.2 µm). In terms of rheological parameters, Formulation C and Uriage^®^ Xémose presented higher apparent viscosity values, 23.7 Pa.s and 25.9 Pa.s, respectively, and showed a similar viscoelastic profile to Shiseido^®^ primer. Uriage^®^ Xémose and Formulation C displayed very similar textural parameters, which suggests that Formulation C has adequate sensory characteristics for a face cream. Other polysaccharides presented similar behavior, as demonstrated by Miastkowska et al. [55], that developed a nanoemulsion gel containing 1.0 wt.% hyaluronic acid displaying a lower apparent viscosity (22.43 Pa.s at 1.0 s^−1^) when compared to tested market preparations (e.g., 55.58 Pa.s at 1.0 s^−1^) but higher spreadability. On the other hand, Danila et al. [56] found that higher concentrations of xanthan gum in the formulation (0.2–1.0 wt.%) resulted in higher apparent viscosity values [56]. In general, Formulation C seems to have suitable physical characteristics to be used in cosmetic products, being, in some cases, equal or superior to the tested commercial products.

## 3. Materials and Methods

### 3.1. Materials

*Olea europaea* (olive) fruit oil was purchased from a local market. Olive oil is an anti-aging ingredient indicated for dermatology applications due to its acidity, antioxidant activity, and soothing effect [9,24], preventing, for example, the appearance of stretch marks [25]. α-tocopherol (vitamin E) was acquired from Sigma-Aldrich (Munich, Germany). α-tocopherol is widely used as a cosmetic antioxidant ingredient, presenting an active role in anti-aging mechanisms, and acting as a coadjutant in atopic dermatitis and melanoma treatments [57,58]. Olive oil and α-tocopherol at concentrations of 20–30 wt.% and 1.0–5.0 wt.%, respectively, were used previously to prepare FucoPol-based emulsions [14].

FucoPol was produced by the bioreactor (Sartorius, Göttingen, Germany) cultivation (10 L) of *Enterobacter* A47 (DSM 23139) on glycerol-supplemented medium, as previously described [18], and extracted from the cultivation broth by ultrafiltration with a 30 kDa membrane, according to the method previously described [17]. FucoPol was composed of 40 mol% fucose, 29 mol% glucose, 24 mol% galactose, and 7.0 mol% glucuronic acid, with a total acyl group content of 11.6 wt.%. The sample had protein and inorganic salt contents of 8.2 wt.% and 4.0 wt.%, respectively.

Other ingredients that were selected for the emulsions’ formulation were cetyl alcohol, glycerin, triethanolamine (TEA), and methyl paraben. Cetyl alcohol is a long-chain alcohol [12] commonly used in cosmetics at concentrations of 0.1–5.0 wt.% [59], with no toxic effects, as a co-emulsifier [60], surfactant [61], thickener [62], and opacifying agent [63]. As a co-emulsifier, a cetyl alcohol concentration higher than 2.0% should be avoided to prevent a soaping effect [64]. Glycerin, responsible for the improvement of skin’s smoothness and moisture [65], is used as a humectant in cosmetics at variable concentrations: 10% in face/neck products; 5.0% in body/hand products; 3.3% in moisturizing products [66]. TEA is used in cosmetics as a pH adjuster [67] and used in personal care products at concentrations between 0.0002% and 19% [64,67,68,69,70]. Methyl paraben, a safe preservative ingredient found in most cosmetics products [38], can be used singly or in combination to enhance the antimicrobial effect, at concentrations below 0.3% [71,72], being normally a non-irritating and non-sensitizing ingredient [71]. Methyl paraben and cetyl alcohol were acquired from Sigma-Aldrich (Munich, Germany). TEA was acquired from Acros Organics B.V.B.A. (Geel, Belgium), and glycerin was acquired from Honeywell (Seelze, Germany).

The commercial emulsifiers stearic acid and Sepigel^®^ 305, acquired from Sigma-Aldrich (Munich, Germany) and from SEPPIC (Courbevoie, France), respectively, were tested for comparison with FucoPol. Stearic acid is a solid saturated fatty acid [73] commonly present in cosmetic formulations (92–96% of products) [74,75] in concentrations between 1.0–25% for moisturizing skin care applications [76]. Sepigel^®^ 305 is a synthetic hydrophilic polymer that provides increased viscosity and stability in cosmetic formulations [14,77].

### 3.2. Factorial Design of Experiments

Response surface methodology (RSM) [78] was applied to determine the best conditions for the development of cosmetic formulations stabilized with FucoPol. A three-factor central composite design (CCD) analyzed the effect of independent variables (Table 5): FucoPol (A: 0.0–1.5 wt.%); cetyl alcohol (B: 0.0–1.5 wt.%), and glycerin (C: 1.0–3.0 wt.%).

The mathematical relationship between the independent variables can be approximated by the second-order polynomial model equation:

(1) $$Y=\beta_{0}+\sum_{i=1}^{n}\beta_{i}x_{i}+\sum_{i=1}^{n}\sum_{j=1}^{n}\beta_{ij}x_{i}x_{j}+\sum_{i=1}^{n}\beta_{ii}x_{i}^{2}\;$$

where *Y* is the predicted response; *x_i_* are the independent variables (*n* = 3). The parameter *β*_0_ is the model constant; *β_i_* are the linear coefficients; *β_ii_* are the quadratic coefficients and *β_ij_* are the cross-product coefficients [14]. A full factorial design of experiments was obtained using the Design-Expert (Design-Expert^®^ software package from Stat-Ease Inc., Minneapolis, MN, USA). The validated model was plotted in a three-dimensional graph, generating a surface response that corresponds to the best emulsification index and apparent viscosity. Analysis of variance (ANOVA) was used to determine the regression coefficients of individual linear, quadratic, and interaction terms.

The emulsions were prepared by heating the oil phase (1.63 g) comprising olive oil (30 wt.%), cetyl alcohol (0.0–2.0 wt.%) and α-tocopherol (2.5 wt.%), and the aqueous phase (3.37 g) comprising FucoPol (0.0–2.0 wt.%), glycerin (1.0–3.0 wt.%), TEA (0.5 wt.%), and methyl paraben (0.02 wt.%) at 75 °C in a recirculated heated water bath Thermomix^®^ ME (B.Braun, Melsungen, Germany). The mixtures were emulsified by manual agitation for 40 s, followed by vortex agitation for 10 s. The emulsification index (EI, %) was determined by the following equation [17]:

(2) $$\mathrm{EI}\;=\frac{h_{e}}{h_{T}}\times 100$$

where h_e_ (mm) is the emulsion layer height and h_T_ (mm) is the overall height of the mixture after emulsification.

### 3.3. Preparation of Fucopol-Based Emulsion Formulations

Six formulations were prepared according to Table 6, including three formulations based on FucoPol as the main emulsifier (formulations A, B, and C) and three formulations based on stearic acid and/or Sepigel^®^ 305 as emulsifier agents (formulations D, E, and F). Formulation A was prepared with FucoPol as the sole emulsifier, while formulation B additionally contained 1.5 wt.% cetyl alcohol as the co-emulsifier. Formulation C was similar to formulation B but 3.0 wt.% glycerin was added to it as an emollient (Table 6). Three other formulations were developed using synthetic emulsifying agents and compared with the FucoPol-based formulations. Formulations D and F were similar to formulation C, but FucoPol was replaced by stearic acid as the main emulsifier at two concentrations, namely, 5.0 and 1.5 wt.%, respectively. Formulation E was similar to formulation F but the co-emulsifier cetyl alcohol was replaced by Sepigel^®^ 305 (1.5 wt.%).

For preparing the emulsions, the oil phase (32.5 g) and the aqueous phase (67.5 g) were heated at 75 °C in a recirculated heated water bath Thermomix^®^ ME (B.Braun, Melsungen, Germany). The emulsification was performed by slowly adding the oil phase to the aqueous phase and mixing with a shear rate of about 11,000 rpm (IKA T25 easy clean digital ULTRA TURRAX, Staufen, Germany), for 3 min, followed by manual continuous stirring until room temperature was attained [3]. All formulations were prepared in batches of 100 g.

### 3.4. Formulations’ Characterization

#### 3.4.1. Physicochemical Properties

The organoleptic (color, odor, appearance) and macroscopic appearance of each formulation were visually analyzed. The EI was evaluated during the storage period (t = 1, 3, 7, 30, 60 days) as described in Section 2.3.

The pH and conductivity were determined by dispersing the formulation sample in deionized water (10%, *w*/*w*) [34,79,80]. The emulsion type was determined as described by Baptista et al. [14], by placing a droplet of the test emulsion onto Whatman™ filter paper (0.2 µm, GE Healthcare Life Sciences, Munich, Germany) and observing the droplet’s dispersion. For the microscopic observation, 10 µL of the sample was stained with 1% (*v*/*v*) Nile Blue A (Sigma-Aldrich, Darmstadt, Germany) and observed in a Zeiss Imager D2 epifluorescence microscope (Carl Zeiss, Oberkochen, Germany), with a magnification of 100×, through ZEN lite software (Carl Zeiss, Oberkochen, Germany). The physical stability was evaluated by centrifuging 1 g of the sample, at 4800 rpm, for 30 min [81].

Dynamic Light Scattering (DLS) was performed to determine the average particle size, the polydispersity index (PI), and the Zeta Potential, using a nanoPartica SZ-100V2 series (Horiba, Lier, Belgium) with a laser of 532 nm and controlling temperature with a Peltier system (25 °C). DLS measurements were performed by diluting the samples (1:10, *w*/*w*) in a disposable cell with a scattering angle equal to 90°. Cumulants statistics data analysis was performed to determine the hydrodynamic size and polydispersity. Zeta Potential measurements were performed in a graphite electrode cell with a 173° scattering angle [20].

#### 3.4.2. Viscoelastic Properties

The formulations’ rheological properties were studied using an MCR 92 modular compact rheometer (Anton Paar, Graz, Austria), equipped with a CP35-2 cone-plate sensor system (angle 2°, diameter 35 mm) and a P-PTD 200/AIR Peltier plate to keep the measurement temperature constant at 25 °C. Dynamic viscosity measurements were performed at shear rates between 0.01 and 1000 s^−1^. Frequency sweep analysis was performed at frequencies ranging from 0.01 to 10 Hz, for a constant strain of 0.1–1.0% that was well within the linear viscoelastic limit evaluated through preliminary amplitude sweep tests [14].

#### 3.4.3. Texture Analysis

The firmness, consistency, cohesiveness, and adhesivity of the attained formulations were determined using a texture analyzer (TMS-Pro, Food Technology Corporation, Sterling, VA, USA) equipped with a 10 N load cell (Mecmesin, Sterling, VA, USA). The sample was placed in a female conic holder and compressed at 11 mm of depth (which represented a sample deformation of around 70%); this procedure was done twice by a male conic probe at a speed of 2 mm/s [14].

## 4. Conclusions

This study demonstrates FucoPol’s suitability for the development of emulsified formulations with good physical and chemical properties for their utilization as cosmetic creams. The fucose-rich biopolymer has shown to possess great potential to replace stearic acid as an emulsifier, resulting in emulsions with similar/better stability, viscosity, firmness, spreadability, and droplet size, which were also shown to be comparable to commercial creams. Although further tests must be done to fine-tune the formulations, the results obtained substantiate the relevance of FucoPol in the development of topical formulations.

## Figures and Tables

**Table 1:** Central composite design (CCD) with studied variables (A: FucoPol, B: Cetyl alcohol, C: Glycerin), experimental values E24 (emulsification index measured at 24 h) and *ƞ* (apparent viscosity measured at a shear rate of 0.1 s^−1^). Organoleptic characteristics and physical stability (centrifugation test) of experiments. SCV—Smooth, creamy, viscous, OS—Olive smell, YW—yellowish white.

Run	FucoPol, A (wt.%)	Cetyl Alcohol, B (wt.%)	Glycerin, C (wt.%)	E24 (%)	*Ƞ* (Pa.s)	Organoleptic Characteristics			Physical Stability
						Color	Appearance	Odor	
1	1.50	0.00	3.00	95	206	YW	SCV	OS	Yes
2	0.75	0.75	2.00	100	20	YW	SCV	OS	No
3	1.50	0.00	1.00	98	206	YW	SCV	OS	Yes
4	1.50	1.50	3.00	98	249	YW	SCV	OS	Yes
5	0.75	0.75	2.00	100	52	YW	SCV	OS	No
6	1.50	1.50	1.00	98	244	YW	SCV	OS	Yes
7	0.00	0.00	1.00	0.00	-	-	-	-	-
8	0.00	1.50	3.00	0.00	-	-	-	-	-
9	0.00	1.50	1.00	0.00	-	-	-	-	-
10	0.75	0.75	2.00	100	21	YW	SCV	OS	No
11	0.00	0.00	3.00	0.00	-	-	-	-	-

**Table 2:** Apparent viscosity (*ƞ*, measured at 2.30 s^−1^) and viscoelastic parameters (G′, G″) at room temperature (~20 °C) for the emulsified formulations (A, B, C, D, E, F), for different storage times. G′-storage/elastic modulus and G″-loss/viscous modulus at *f* = 0.1 Hz.

Time (Days)	A			B			C		
	*Ƞ* (Pa.s)	G′ (Pa)	G″ (Pa)	*Ƞ* (Pa.s)	G′ (Pa)	G″ (Pa)	*Ƞ* (Pa.s)	G′ (Pa)	G″ (Pa)
1	8.72	64.8	36.6	19.5	94.6	55.7	34.3	137	58.8
3	7.91	41.8	29.8	19.7	53.2	38.0	27.2	87.1	43.4
7	7.97	23.8	22.8	19.6	78.8	48.5	30.6	151	59.2
30	6.42	21.1	19.3	16.0	58.2	36.5	23.7	82.5	40.1
60	6.10	11.8	14.9	15.5	35.1	28.2	22.4	56.2	35.2
**Time** **(Days)**	**D**			**E**			**F**		
	***Ƞ*** **(Pa.s)**	**G′** **(Pa)**	**G″** **(Pa)**	***Ƞ*** **(Pa.s)**	**G′** **(Pa)**	**G″** **(Pa)**	***Ƞ*** **(Pa.s)**	**G′** **(Pa)**	**G″** **(Pa)**
1	8.75	949	249	59.6	1329	94.2	6.19	286	75.6
3	8.30	708	198	54.9	1219	84.7	8.72	267	72.7
7	8.97	573	146	54.0	1349	97.7	7.51	269	74.6
30	6.98	492	188	36.0	1217	98.9	5.83	284	92.4
60	6.57	483	125	38.8	1262	93.9	7.69	269	80.1

**Table 3:** Numerical values of the textural parameters for formulations tested of t = 1 and t = 60 at room temperature.

Formulation	Time (Days)	Textural Parameters			
		Firmness (N)	Consistency (mJ)	Cohesiveness (N)	Adhesiveness (mJ)
A	1	0.064	0.261	0.741	0.244
	60	0.029	0.119	0.970	0.133
B	1	0.162	0.505	0.925	0.467
	60	0.088	0.198	0.921	0.266
C	1	0.194	0.387	1.034	0.387
	60	0.047	0.160	1.004	0.129
D	1	0.115	0.445	0.931	0.338
	60	0.067	0.225	0.891	0.169
E	1	0.136	0.504	0.852	0.499
	60	0.086	0.231	1.087	0.188
F	1	0.097	0.319	0.976	0.317
	60	0.049	0.273	0.844	0.126

**Table 4:** Rheological parameters and textural parameters of commercial products tested. Apparent viscosity (*ƞ*, measured at 2.30 s^−1^) and viscoelastic parameters (G′, G″) measured at room temperature (~20 °C). G′-storage/elastic modulus and G″-loss/viscous modulus, at f = 0.1 Hz.

Product	pH	Conductivity (µS/cm)	Droplet Size (µm)	Rheological Parameters			Textural Parameters			
				*Ƞ* (Pa.s)	G′ (Pa)	G″ (Pa)	Firmness (N)	Consistency (mJ)	Cohesiveness (N)	Adhesiveness (mJ)
Cien^®^ Body lotion	5.78	739	15.9	12.1	800	156	0.056	0.329	0.832	0.273
Uriage^®^ Pruriced	7.95	727	13.5	14.2	25.1	9.27	0.062	0.224	0.980	0.245
Shiseido^®^ Primer	8.17	257	22.0	6.93	207	57.6	0.068	0.198	0.897	0.193
Sephora^®^ Hand cream	8.18	105	27.9	73.4	5575	625	0.190	0.699	0.931	0.471
Uriage^®^ Xémose	6.68	510	19.0	25.9	25.1	9.27	0.130	0.543	1.049	0.449
Formulation C	6.30	138	24.2	23.7	203	68.7	0.047	0.117	0.943	0.192

**Table 5:** Independent variables and their coded levels used in the RSM.

Independent Variables	Coded Variables	Factor Level		
		−1	0	1
FucoPol (wt.%) Cetyl alcohol (wt.%)	A	0.00	0.75	1.50
	B	0.00	0.75	1.50
Glycerin (wt.%)	C	1.00	2.00	3.00

**Table 6:** Cosmetic formulation composition (wt.%). q.s.–quantity sufficient.

INCI Name	Function	Concentration (wt.%)					
Aqueous Phase		A	B	C	D	E	F
Water	Solvent	q.s.	q.s.	q.s.	q.s.	q.s.	q.s.
FucoPol	Emulsifier agent	1.5	1.5	1.5	-	-	-
Sepigel^®^ 305	Emulsifier agent	-	-	-	-	1.5	-
Glycerin	Emollient/humectant	-	-	3	3	3	3
Methyl paraben	Preservative	0.02	0.02	0.02	0.02	0.02	0.02
TEA	pH regulator	0.5	0.5	0.5	0.5	0.5	0.5
**Oil phase**							
Cetyl alcohol	Co-emulsifier agent	-	1.5	1.5	1.5	-	1.5
Stearic acid	Emulsifier agent	-	-	-	5	1.5	1.5
*Olea europaea* (Olive) fruit oil	Oil, dispersed phase	30	30	30	30	30	30
α-tocopherol	Antioxidant	2.5	2.5	2.5	2.5	2.5	2.5

## Data Availability

Data will be available upon request.

## Supplementary Materials

The following supporting information can be downloaded at: https://www.mdpi.com/article/10.3390/molecules27227759/s1, Figure S1: Three-dimensional response (A: E24; B: ƞ) surface plot showing the interactive effects of different ingredients on the O/W emulsion. (a) FucoPol and cetyl alcohol (wt.%) with glycerin fixed at 2.0 wt.%, (b) FucoPol and glycerin (wt.%) with cetyl alcohol fixed at 0.75 wt.%, (c) cetyl alcohol and glycerin (wt.%) with FucoPol fixed at 0.75 wt.%; Figure S2: Formulations A, B, C, D, E, F during the storage period: t = 1, t = 3, t = 7, t = 30, and t = 60 (days); Figure S3: Physical stability evaluation test by centrifugation for formulations A, B, C, D, E, F during the storage period: t = 1, t = 3, t = 7, t = 30, and t = 60 (days); Figure S4: Mechanical spectra for formulations A (a), B (b), C (c), D (d), E (e), and F (f) during the storage period; t = 1 day (orange), t = 3 days (blue), t = 7 days (green), t = 30 days (yellow), and t = 60 days (gray). G′ (open triangle) and G″ (closed triangle).

## References

[1] Baptista S., Freitas F., Oliveira J., Radhouani H., Reis R.L. (2021). Bacterial Polysaccharides: Cosmetic Applications. Polysaccharides of Microbial Origin.

[2] Semenzato A., Costantini A., Baratto G. (2015). Green Polymers in Personal Care Products: Rheological Properties of Tamarind Seed Polysaccharide. Cosmetics.

[3] Bom S., Fitas M., Martins A.M., Pinto P., Ribeiro H.M., Marto J. (2020). Replacing Synthetic Ingredients by Sustainable Natural Alternatives: A Case Study Using Topical O/W Emulsions. Molecules.

[4] Freitas F., Alves V., Reis M.A.M. (2014). Bacterial Polysaccharides: Production and Applications in Cosmetic Industry. Polysaccharides.

[5] Nadzir M.M., Nurhayati R.W., Idris F.N., Nguyen M.H. (2021). Biomedical Applications of Bacterial Exopolysaccharides: A Review. Polymers.

[6] Li Z., Dai L., Wang D., Mao L., Gao Y. (2018). Stabilization and Rheology of Concentrated Emulsions Using the Natural Emulsifiers *Quillaja* Saponins and Rhamnolipids. J. Agric. Food Chem..

[7] Tang Q., Huang G. (2022). Improving Method, Properties and Application of Polysaccharide as Emulsifier. Food Chem..

[8] Gu R., Li C., Shi X., Xiao H. (2022). Naturally Occurring Protein/Polysaccharide Hybrid Nanoparticles for Stabilizing Oil-in-Water Pickering Emulsions and the Formation Mechanism. Food Chem..

[9] Santamaria-Echart A., Fernandes I.P., Silva S.C., Rezende S.C., Colucci G., Dias M.M., Barreiro M.F., Prieto M.A., Otero P. (2022). New Trends in Natural Emulsifiers and Emulsion Technology for the Food Industry. Natural Food Additives.

[10] Dapueto N., Troncoso E., Mella C., Zúñiga R.N. (2019). The Effect of Denaturation Degree of Protein on the Microstructure, Rheology and Physical Stability of Oil-in-Water (O/W) Emulsions Stabilized by Whey Protein Isolate. J. Food Eng..

[11] Burgos-Díaz C., Wandersleben T., Marqués A.M., Rubilar M. (2016). Multilayer Emulsions Stabilized by Vegetable Proteins and Polysaccharides. Curr. Opin. Colloid Interface Sci..

[12] Caritá A.C., Resende de Azevedo J., Vinícius Buri M., Bolzinger M.A., Chevalier Y., Riske K.A., Ricci Leonardi G. (2021). Stabilization of Vitamin C in Emulsions of Liquid Crystalline Structures. Int. J. Pharm..

[13] Melanie H., Taarji N., Zhao Y., Khalid N., Neves M.A., Kobayashi I., Tuwo A., Nakajima M. (2020). Formulation and Characterization of O/W Emulsions Stabilized with Modified Seaweed Polysaccharides. Int. J. Food Sci. Technol..

[14] Baptista S., Pereira J.R., Gil C.V., Torres C.A.V., Reis M.A.M., Freitas F. (2022). Development of Olive Oil and α -Tocopherol Containing Emulsions Stabilized by FucoPol: Rheological and Textural Analyses. Polymers.

[15] Medina-Torres L., Calderas F., Sanchez-Olivares G., Nuñez-Ramirez D.M. (2014). Rheology of Sodium Polyacrylate as an Emulsifier Employed in Cosmetic Emulsions. Ind. Eng. Chem. Res..

[16] Calvo F., Gómez J.M., Ricardez-Sandoval L., Alvarez O. (2020). Integrated Design of Emulsified Cosmetic Products: A Review. Chem. Eng. Res. Des..

[17] Baptista S., Torres C.A.V., Sevrin C., Grandfils C., Reis M.A.M., Freitas F. (2022). Extraction of the Bacterial Extracellular Polysaccharide FucoPol by Membrane-Based Methods: Efficiency and Impact on Biopolymer Properties. Polymers.

[18] Freitas F., Alves V.D., Gouveia A.R., Pinheiro P., Torres C.A.V., Grandfils C., Reis M.A.M. (2014). Controlled Production of Exopolysaccharides from *Enterobacter* A47 as a Function of Carbon Source with Demonstration of Their Film and Emulsifying Abilities. Appl. Biochem. Biotechnol..

[19] Freitas F., Alves V.D., Torres C.A.V., Cruz M., Sousa I., João M., Ramos A.M., Reis M.A.M. (2011). Fucose-Containing Exopolysaccharide Produced by the Newly Isolated *Enterobacter* strain A47 DSM 2313. Carbohydr. Polym..

[20] Antunes S.A.D.C.S. (2018). Biological Conversion of Industrial By-Products/Wastes into Value-Added Bacterial Exopolysaccharides. Ph.D. Thesis.

[21] Concórdio-Reis P., Pereira C.V., Batista M.P., Sevrin C., Grandfils C., Marques A.C., Fortunato E., Gaspar F.B., Matias A.A., Freitas F. (2020). Silver Nanocomposites Based on the Bacterial Fucose-Rich Polysaccharide Secreted by *Enterobacter* A47 for Wound Dressing Applications: Synthesis, Characterization and in Vitro Bioactivity. Int. J. Biol. Macromol..

[22] Guerreiro B.M., Freitas F., Lima J.C., Silva J.C., Reis M.A.M. (2021). Photoprotective Effect of the Fucose-Containing Polysaccharide FucoPol. Carbohydr. Polym..

[23] Guerreiro B.M., Silva J.C., Lima J.C., Reis M.A.M., Freitas F. (2021). Antioxidant Potential of the Bio-Based Fucose-Rich Polysaccharide FucoPol Supports Its Use in Oxidative Stress-Inducing Systems. Polymers.

[24] Torres C.A.V., Ferreira A.R.V., Freitas F., Reis M.A.M., Coelhoso I., Sousa I., Alves V.D. (2015). Rheological Studies of the Fucose-Rich Exopolysaccharide FucoPol. Int. J. Biol. Macromol..

[25] Khuri A.I., Mukhopadhyay S. (2010). Response Surface Methodology. Wiley. Interdiscip. Rev. Comput. Stat..

[26] Jaslina N.F., Faujan N.H., Mohamad R., Ashari S.E. (2020). Effect of Addition of PVA/PG to Oil-in-Water Nanoemulsion Kojic Monooleate Formulation on Droplet Size: Three-Factors Response Surface. Cosmetics.

[27] Yu L., Li S., Stubbs L.P., Lau H.C. (2021). Effects of Salinity and pH on the Stability of Clay-Stabilized Oil-in-Water Pickering Emulsions. SPE J..

[28] Dammak M., Hlima H., Smaoui S., Fendri I., Michaud P., Ayadi M.A., Abdelkafi S. (2022). Conception of an Environmental Friendly O/W Cosmetic Emulsion from Microalgae. Environ. Sci. Pollut. Res..

[29] Dąbrowska M., Nowak I. (2021). Lipid Nanoparticles Loaded with Selected Iridoid Glycosides as Effective Components of Hydrogel Formulations. Materials.

[30] Martínez-Pla J.J., Martín-Biosca Y., Sagrado S., Villanueva-Camañas R.M., Medina-Hernández M.J. (2004). Evaluation of the PH Effect of Formulations on the Skin Permeability of Drugs by Biopartitioning Micellar Chromatography. J. Chromatogr. A.

[31] Khan B.A., Akhtar N., Khan H., Braga V.A. (2013). Development, Characterization and Antioxidant Activity of Polysorbate Based O/W Emulsion Containing Polyphenols Derived from *Hippophae rhamnoides* and *Cassia fistula*. Braz. J. Pharm. Sci..

[32] Kaur N., Kaur M., Mahajan M., Jain S.K. (2020). Development, Characterization and Evaluation of Nanocarrier Based Formulations of Antipsoriatic Drug “Acitretin” for Skin Targeting. J. Drug Deliv. Sci. Technol..

[33] Masmoudi H., Dréau Y.L., Piccerelle P., Kister J. (2005). The Evaluation of Cosmetic and Pharmaceutical Emulsions Aging Process Using Classical Techniques and a New Method: FTIR. Int. J. Pharm..

[34] Afifah S.N., Azhar S., Ashari S.E., Salim N. (2018). Development of a Kojic Monooleate-Enriched Oil-in-Water Nanoemulsion as a Potential Carrier for Hyperpigmentation Treatment. Int. J. Nanomed..

[35] Kavitake D., Balyan S., Devi P.B., Shetty P.H. (2020). Evaluation of Oil-in-Water (O/W) Emulsifying Properties of Galactan Exopolysaccharide from *Weissella confusa* KR780676. J. Food Sci. Technol..

[36] Kavitake D., Balyan S., Devi P.B., Shetty P.H. (2019). Interface between Food Grade Flavour and Water Soluble Galactan Biopolymer to Form a Stable Water-in-Oil-in-Water Emulsion. Int. J. Biol. Macromol..

[37] McClements D.J. (2007). Critical Review of Techniques and Methodologies for Characterization of Emulsion Stability. Crit. Rev. Food Sci. Nutr..

[38] Shkreli R., Terziu R., Memushaj L., Dhamo K. (2022). Formulation and Stability Evaluation of a Cosmetics Emulsion Loaded with Different Concentrations of Synthetic and Natural Preservative. Polymers.

[39] Huber P., Yamashita Y., Maibach H., Lochhead R., Sakamoto K. (2017). Sensory Measurement-Evaluation and Testing of Cosmetic Products. Cosmetic Science and Technology: Theoretical Principles and Applications.

[40] Niu F., Han B., Fan J., Kou M., Zhang B., Feng Z.J., Pan W., Zhou W. (2018). Characterization of Structure and Stability of Emulsions Stabilized with Cellulose Macro/Nano Particles. Carbohydr. Polym..

[41] Estanqueiro M., Conceição J., Amaral M.H., Sousa Lobo J.M. (2014). Characterization, Sensorial Evaluation and Moisturizing Efficacy of Nanolipidgel Formulations. Int. J. Cosmet. Sci..

[42] Karbstein H., Schubert H. (1995). Developments in the Continuous Mechanical Production of Oil-in-Water Macro-Emulsions. Chem. Eng. Process. Process Intensif..

[43] Venkataramani D., Tsulaia A., Amin S. (2020). Fundamentals and Applications of Particle Stabilized Emulsions in Cosmetic Formulations. Adv. Colloid Interface Sci..

[44] Akbari S., Nour A.H. (2018). Emulsion Types, Stability Mechanisms and Rheology: A Review. Int. J. Innov. Res. Sci. Stud..

[45] Gullapalli R.P., Sheth B.B. (1999). Influence of an Optimized Non-Ionic Emulsifier Blend on Properties of Oil-in-Water Emulsions. Eur. J. Pharm. Biopharm..

[46] Dapčević Hadnadev T., Dokić P., Krstonošić V., Hadnadev M. (2013). Influence of Oil Phase Concentration on Droplet Size Distribution and Stability of Oil-in-Water Emulsions. Eur. J. Lipid Sci. Technol..

[47] Bhattacharjee S. (2016). DLS and Zeta Potential—What They Are and What They Are Not?. J. Control. Release.

[48] Dickinson E., Phillips G.O., Williams P.A. (2009). Hydrocolloids and Emulsion Stability. Handbook of Hydrocolloids.

[49] Nunes A., Gonçalves L., Marto J., Martins A.M., Silva A.N., Pinto P., Martins M., Fraga C., Ribeiro H.M. (2021). Investigations of Olive Oil Industry By-Products Extracts with Potential Skin Benefits in Topical Formulations. Pharmaceutics.

[50] Paximada P., Tsouko E., Kopsahelis N., Koutinas A.A., Mandala I. (2016). Bacterial Cellulose as Stabilizer of o/w Emulsions. Food Hydrocoll..

[51] Tafuro G., Costantini A., Baratto G., Francescato S., Busata L., Semenzato A. (2021). Characterization of Polysaccharidic Associations for Cosmetic Use: Rheology and Texture Analysis. Cosmetics.

[52] Douguet M., Picard C., Savary G., Merlaud F., Loubat-bouleuc N., Grisel M. (2017). Spreading Properties of Cosmetic Emollients Use of Synthetic Skin Surface to Elucidate Structural Effect. Colloids Surf. B Biointerfaces.

[53] César F.C.S., Maia Campos P.M.B.G. (2020). Influence of Vegetable Oils in the Rheology, Texture Profile and Sensory Properties of Cosmetic Formulations Based on Organogel. Int. J. Cosmet. Sci..

[54] Savary G., Grisel M., Picard C. (2013). Impact of Emollients on the Spreading Properties of Cosmetic Products: A Combined Sensory and Instrumental Characterization. Colloids Surf. B Biointerfaces.

[55] Miastkowska M., Kulawik-Pióro A., Szczurek M. (2020). Nanoemulsion Gel Formulation Optimization for Burn Wounds: Analysis of Rheological and Sensory Properties. Processes.

[56] Danila E., Moldovan Z., Albu Kaya M.G., Ghica M.V. (2019). Formulation and Characterization of Some Oil in Water Cosmetic Emulsions Based on Collagen Hydrolysate and Vegetable Oils Mixtures. Pure Appl. Chem..

[57] Martinez R.M., Magalhães W.V., Sufi B.D.S., Padovani G., Nazato L.I.S., Velasco M.V.R., Lannes S.C.S., Baby A.R. (2021). Vitamin E-Loaded Bigels and Emulsions: Physicochemical Characterization and Potential Biological Application. Colloids Surf. B Biointerfaces.

[58] Jaiswal P.V., Ijeri V.S., Srivastava A.K. (2001). Voltammetric Behavior of α-Tocopherol and Its Determination Using Surfactant + Ethanol + Water and Surfactant + Acetonitrile + Water Mixed Solvent Systems. Anal. Chim. Acta.

[59] Andersen A.F. (1988). Final Report on the Safety Assessment of Cetearyl Alcohol, Cetyl Alcohol, Isostearyl Alcohol, Myristyl Alcohol, and Behenyl Alcohol. Int. J. Toxicol..

[60] Djuris J., Vasiljevic D., Jokic S., Ibric S. (2014). Application of D-Optimal Experimental Design Method to Optimize the Formulation of O/W Cosmetic Emulsions. Int. J. Cosmet. Sci..

[61] Dastbaz Z., Ashrafizadeh S.N. (2020). Preparation, Stabilization, and Characterization of Polyisobutylene Aqueous Suspension. Colloid. Polym. Sci..

[62] Huynh A., Abou-Dahech M.S., Reddy C.M., O’Neil G.W., Chandler M., Baki G. (2019). Alkenones, a Renewably Sourced, Biobased Wax as an SPF Booster for Organic Sunscreens. Cosmetics.

[63] Martins M.S., Ferreira M.S., Almeida I.F., Sousa E. (2022). Occurrence of Allergens in Cosmetics for Sensitive Skin. Cosmetics.

[64] Cheng Y.S., Lam K.W., Ng K.M., Ko R.K.M., Wibowo C. (2009). An Integrative Approach to Product Development-A Skin-Care Cream. Comput. Chem. Eng..

[65] Motia S., Bouchikhi B., Llobet E., Bari N. (2020). Synthesis and Characterization of a Highly Sensitive and Selective Electrochemical Sensor Based on Molecularly Imprinted Polymer with Gold Nanoparticles Modified Screen-Printed Electrode for Glycerol Determination in Wastewater. Talanta.

[66] Becker L.C., Bergfeld W.F., Belsito D.V., Hill R.A., Klaassen C.D., Liebler D.C., Marks J.G., Shank R.C., Slaga T.J., Snyder P.W. (2019). Safety Assessment of Glycerin as Used in Cosmetics. Int. J. Toxicol..

[67] Fiume M.M., Heldreth B., Bergfeld W.F., Belsito D.V., Hill R.A., Klaassen C.D., Liebler D., Marks J.G., Shank R.C., Slaga T.J. (2013). Safety Assessment of Triethanolamine and Triethanolamine-Containing Ingredients as Used in Cosmetics. Int. J. Toxicol..

[68] Gupta N., Dubey A., Prasad P., Roy A. (2015). Formulation and Evaluation of Herbal Fairness Cream Comprising Hydroalcoholic Extracts of *Pleurotus ostreatus*, *Glycyrrhiza glabra* and *Camellia sinensis*. Pharm. Biosci. J..

[69] Isabella E., Pohan T. (2013). Formulation of Oil-in-Water Cream from Mangosteen (*Garcinia mangostana* L.) Pericarp Extract Preserved by Gamma Irradiation. Atom Indones..

[70] Kusumawati L.A.I., Dewi E.N.A., Xenograf O.C., Rifrianasari K., Hidayat M.A. (2015). Tyrosinase Inhibition Assay and Skin Whitening Cream Formulation of Edamame Extract (Glycine Max). Int. J. Pharmacogn. Phytochem. Res..

[71] Soni M.G., Taylor S.L., Greenberg N.A., Burdock G.A. (2002). Evaluation of the Health Aspects of Methyl Paraben: A Review of the Published Literature. Food Chem. Toxicol..

[72] Matwiejczuk N., Galicka A., Brzóska M.M. (2020). Review of the Safety of Application of Cosmetic Products Containing Parabens. J. Appl. Toxicol..

[73] Gore E., Picard C., Savary G. (2018). Spreading Behavior of Cosmetic Emulsions: Impact of the Oil Phase. Biotribology.

[74] Aziz A.A., Nordin F.N.M., Zakaria Z., Abu Bakar N.K. (2022). A Systematic Literature Review on the Current Detection Tools for Authentication Analysis of Cosmetic Ingredients. J. Cosmet. Derm..

[75] Vidhyalakshmi R., Valli Nachiyar C., Narendra Kumar G., Sunkar S., Badsha I. (2018). Production, Characterization and Emulsifying Property of Exopolysaccharide Produced by Marine Isolate of *Pseudomonas fluorescens*. Biocatal. Agric. Biotechnol..

[76] Liebert M.A. (1987). Final Report on the Safety Assessment of Oleic Acid, Lauric Acid, Palmitic Acid, Myristic Acid, and Stearic Acid. Int. J. Toxicol..

[77] Berenguer D., Sosa L., Alcover M., Sessa M., Halbaut L., Guillén C., Fisa R., Calpena-Campmany A.C., Riera C. (2019). Development and Characterization of a Semi-Solid Dosage Form of Meglumine Antimoniate for Topical Treatment of Cutaneous Leishmaniasis. Pharmaceutics.

[78] Lundstedt T., Seifert E., Abramo L., Thelin B., Nystrom A., Pettersen J., Bergman R. (1998). Experimental Design and Optimization. Chemom. Intell. Lab. Syst..

[79] Favero J.S., Santos V., Weiss-Angeli V., Gomes L.B., Veras D.G., Dani N., Mexias A.S., Bergmann C.P. (2019). Evaluation and Characterization of Melo Bentonite Clay for Cosmetic Applications. Appl. Clay Sci..

[80] Khan B.A., Akhtar N., Menaa A., Menaa F. (2015). A Novel *Cassia fistula* (L.)-Based Emulsion Elicits Skin Anti-Aging Benefits in Humans. Cosmetics.

[81] Semenzato A., Costantini A., Meloni M., Maramaldi G., Meneghin M., Baratto G. (2018). Formulating O/W Emulsions with Plant-Based Actives: A Stability Challenge for an Effective Product. Cosmetics.

