# Supplementary material for: Formulation of the Polysaccharide FucoPol into Novel Emulsified Creams with Improved Physicochemical Properties
Source: Molecules. 2022 Nov 10;27(22):7759. doi: 10.3390/molecules27227759 (PMC9695255; doi:10.3390/molecules27227759)
Supplement: Supplementary file 1 [file molecules-27-07759-s001.zip › molecules-1925403-supplementary.pdf]

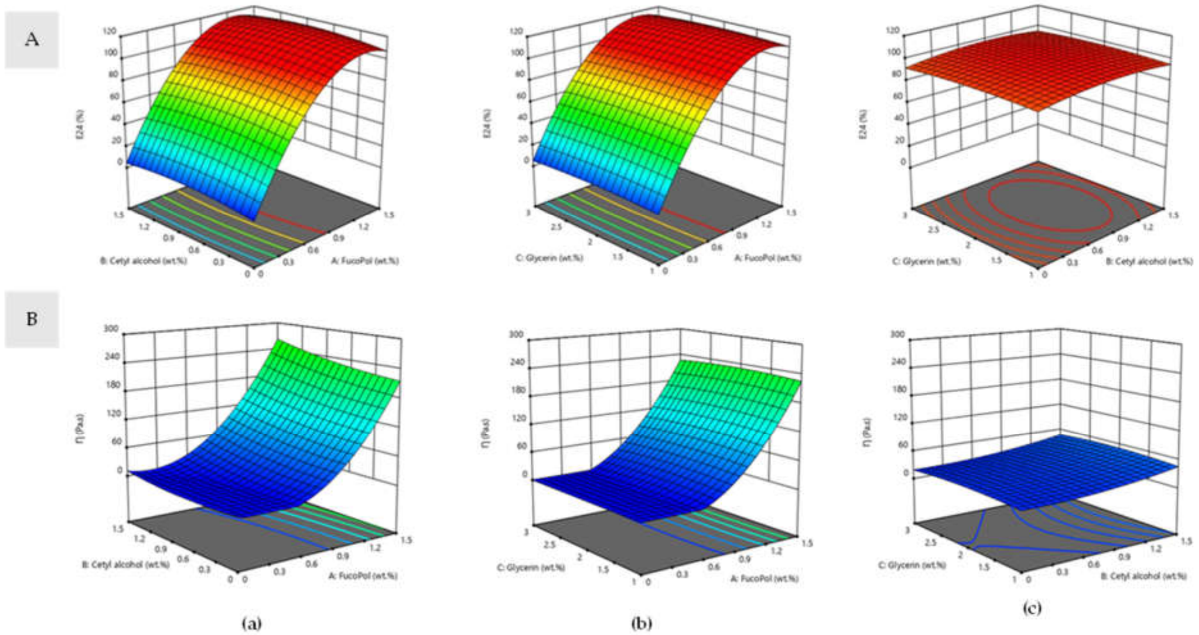

Figure S1. Three-dimensional response (A: E24; B: η) surface plot showing the interactive effects of different ingredients on the O/W emulsion. (**a**) FucoPol and cetyl alcohol (wt.%) with glycerin fixed at 2.0 wt.%, (**b**) FucoPol and glycerin (wt.%) with cetyl alcohol fixed at 0.75 wt.%, (**c**) cetyl alcohol and glycerin (wt.%) with FucoPol fixed at 0.75 wt.%.

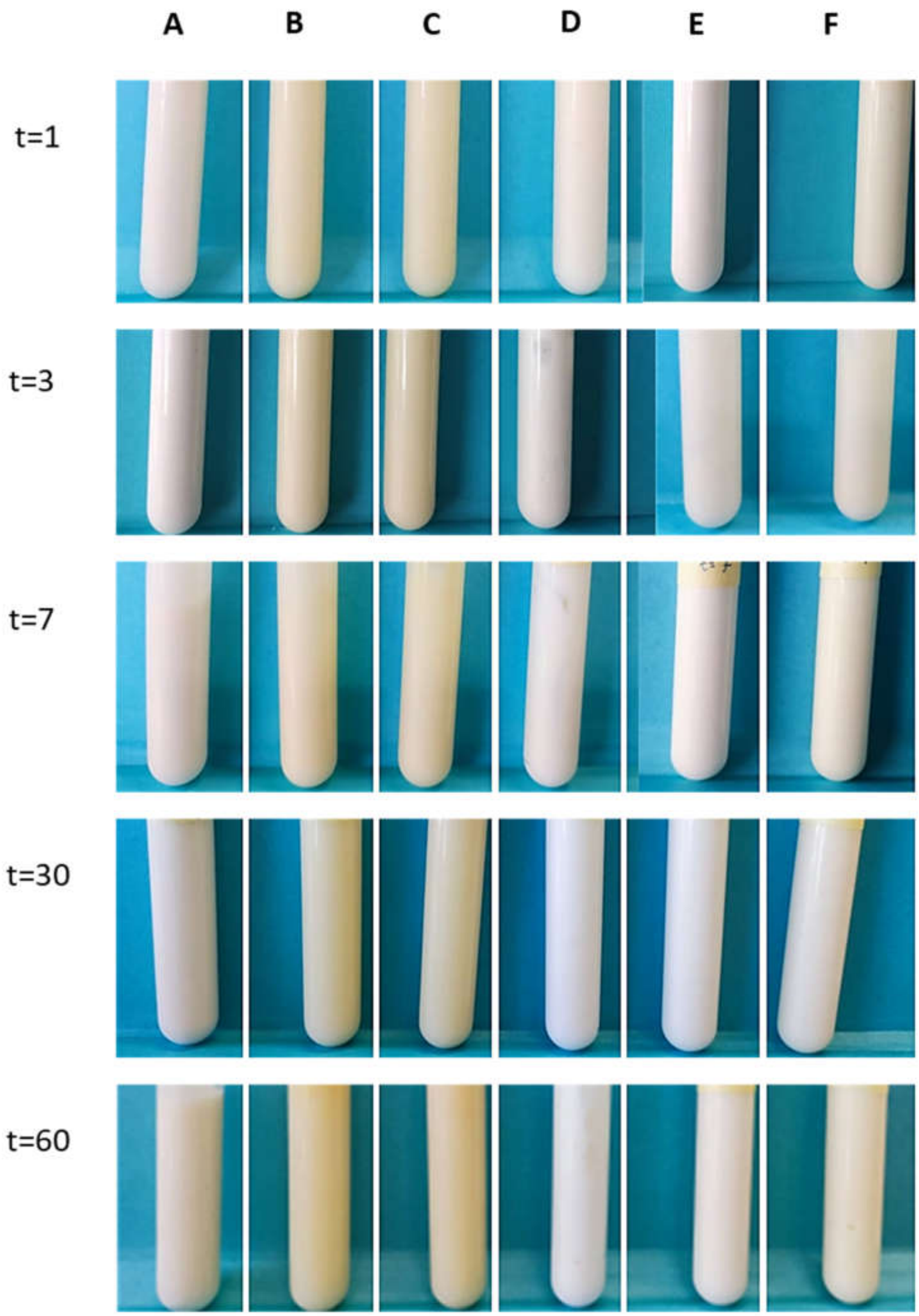

Figure S2. Emulsification index (EI=100%) for formulations A, B, C, D, E, F during the storage times: t=1, t=3, t=7, t=30, and t=60.

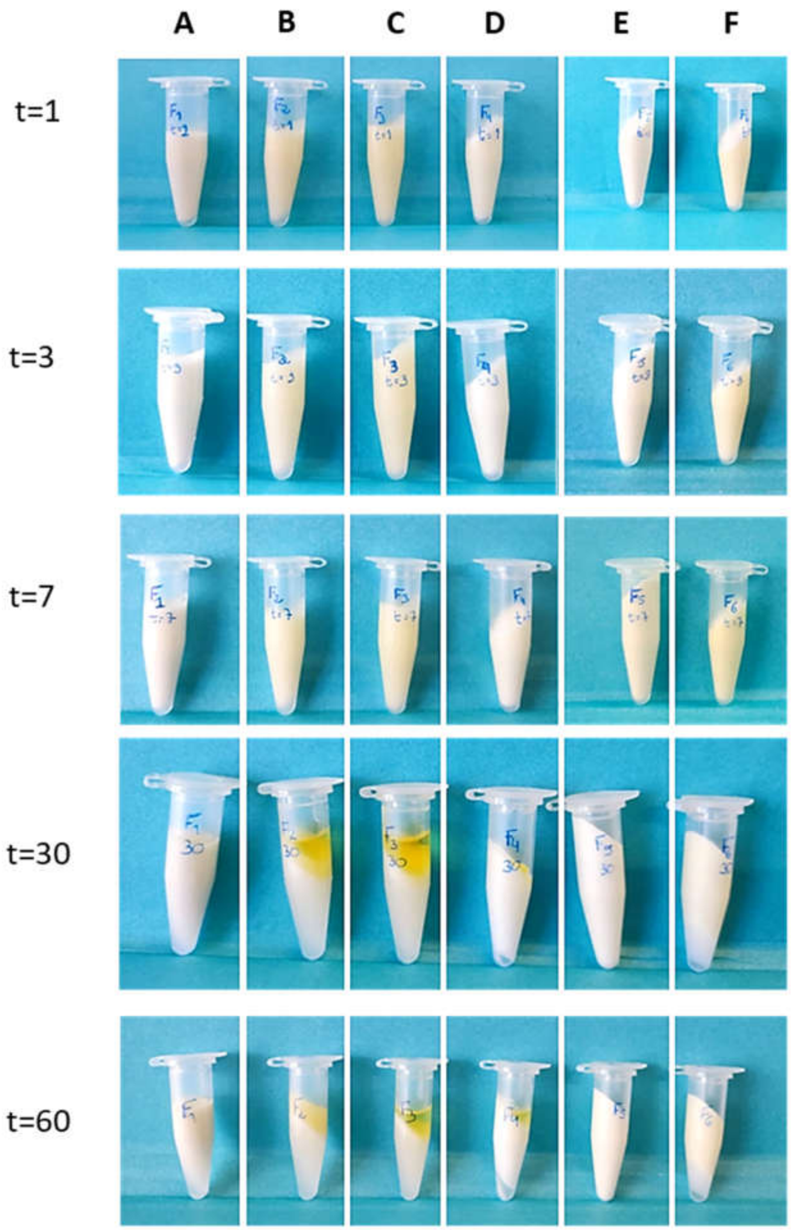

Figure S3. Centrifugation test for formulations A, B, C, D, E, F during the storage times: t=1, t=3, t=7, t=30, and t=60.

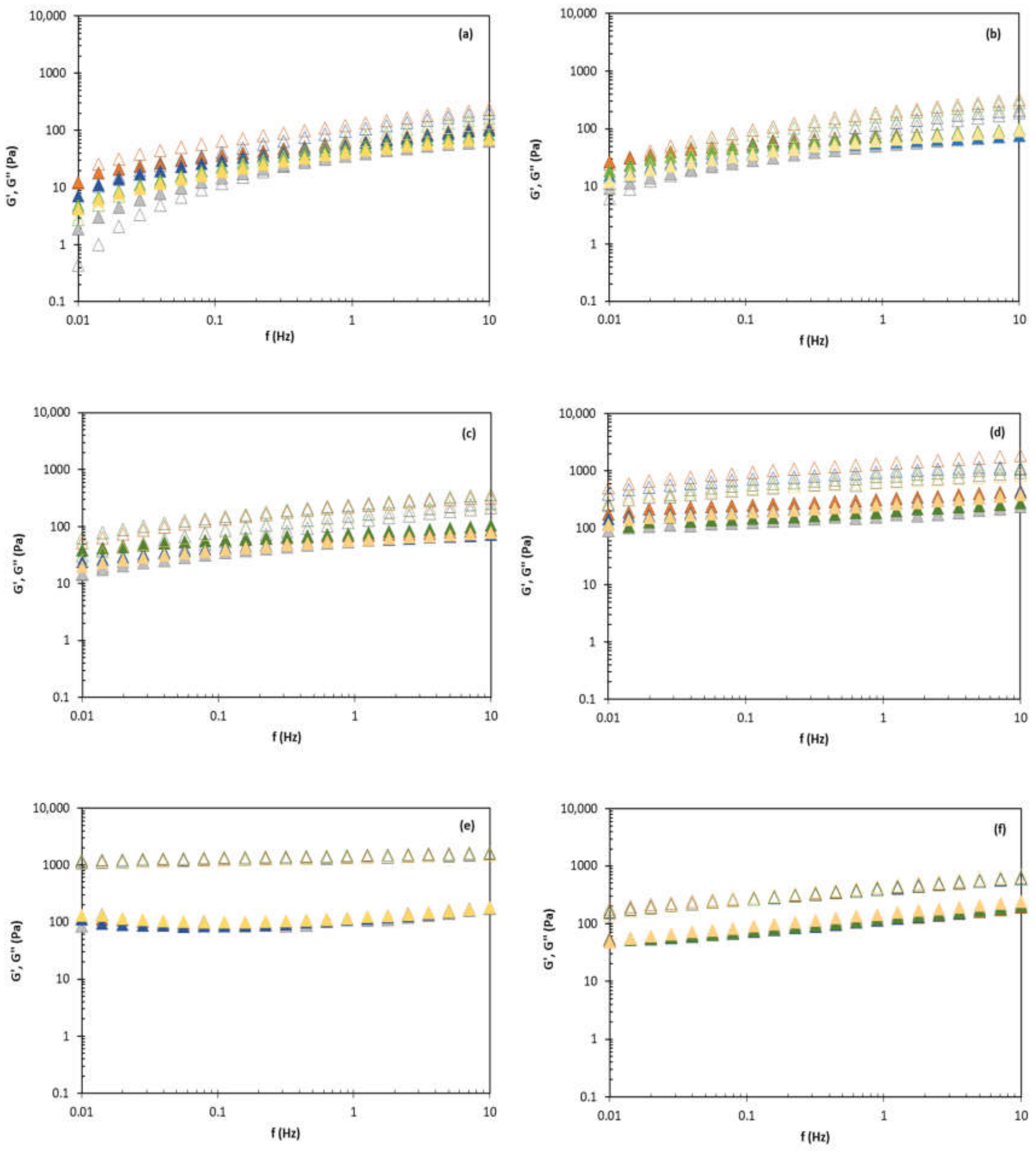

Figure S4. Mechanical spectrum for formulations A (**a**), B (**b**), C (**c**), D (**d**), E (**e**), and F (**f**) during the storage times; t=1 (orange), t=3 (blue), t=7 (green), t=30 (yellow), and t=60 (gray). G′ (open triangle) and G′′ (closed triangle).
